# Learning organisation survey - an assessment of perceptions towards organisational learning

**DOI:** 10.1186/2197-425X-3-S1-A861

**Published:** 2015-10-01

**Authors:** S Kumar, T Leary, S Hutchinson

**Affiliations:** Norfolk & Norwich University Hospitals, Norwich, United Kingdom

## Introduction

Intensive care units are complex working environments, which are constantly assessed and reviewed against national standards. Hence it is more important to constantly self-evaluate and improve quality of care and clinical outcomes. The concept of organisational learning is adopted by the health care industry [1] from the commercial industry, which has benefitted from increased productivity and performance. It is hypothesized that by creating a culture of organisational learning, quality of care and outcomes could be bettered. As a strategy to develop the organisational learning, we are introducing in-situ simulation in our intensive care unit. Creating a supportive learning environment, incorporating concrete learning processes and practices, and a leadership reinforcing the learning, form the foundation blocks of the organisational learning [2].

## Objectives

To assess the current baseline perceptions and deficiencies in our unit, with respect to the building blocks of organisational learning. We aim to incorporate the deficiencies identified into the instructional design of the simulation scenarios and also in the governance structure of the intensive care unit.

## Methods

We conducted an anonymous online survey of 40 questions. The questionnaire was modified for the healthcare environment, and used experienced staff to check construct and face validity. 50 members of staff with varying degree of expertise and experience responded, which includes doctors, nurses, pharmacists, healthcare assistants, and physiotherapists.

## Results

50% felt they were over-stressed when they are at work and 40% felt that little time was spent on improvement, reflection and importantly, it affected good clinical care. 40% felt that their differences in opinion were not valued. 40% felt not enough time and resources were dedicated to teaching and learning. 25% felt that the unit resisted new ideas and innovations and also lacked processes for experimentation. 25% felt that senior staff members showed poor leadership by criticising and discouraging different views. 20% felt uncomfortable to voice their opinions.

## Conclusions

The results highlights only the main negative perceptions in our intensive care unit, but about two-third respondents felt positive about the learning environment in the unit. We aim to improve training provision and reflection by regular in-situ simulation sessions. The no blame culture, openness to others views and leadership qualities will be incorporated into the instructional design of the scenarios and will be stressed in reflection and feedback process. Organisational learning is an ongoing process with lifelong learning, when assessed and strategies incorporated, logically will lead to better quality of care.
